# Improvement of genomic prediction in advanced wheat breeding lines by including additive-by-additive epistasis

**DOI:** 10.1007/s00122-021-04009-4

**Published:** 2022-01-01

**Authors:** Miguel Angel Raffo, Pernille Sarup, Xiangyu Guo, Huiming Liu, Jeppe Reitan Andersen, Jihad Orabi, Ahmed Jahoor, Just Jensen

**Affiliations:** 1 Center for Quantitative Genetics and Genomics, Aarhus University, Tjele, Denmark; 2 Nordic Seed A/S, Galten, Denmark; 3 Department of Plant Breeding, The Swedish University of Agricultural Sciences, Uppsala, Sweden

## Abstract

**Key message:**

Including additive and additive-by-additive epistasis in a NOIA parametrization did not yield orthogonal partitioning of genetic variances, nevertheless, it improved predictive ability in a leave-one-out cross-validation for wheat grain yield.

**Abstract:**

Additive-by-additive epistasis is the principal non-additive genetic effect in inbred wheat lines and is potentially useful for developing cultivars based on total genetic merit; nevertheless, its practical benefits have been highly debated. In this article, we aimed to (i) evaluate the performance of models including additive and additive-by-additive epistatic effects for variance components (VC) estimation of grain yield in a wheat-breeding population, and (ii) to investigate whether including additive-by-additive epistasis in genomic prediction enhance wheat grain yield predictive ability (PA). In total, 2060 sixth-generation (F_6_) lines from Nordic Seed A/S breeding company were phenotyped in 21 year-location combinations in Denmark, and genotyped using a 15 K-Illumina-BeadChip. Three models were used to estimate VC and heritability at plot level: (i) “I-model” (baseline), (ii) “I + G_A_-model”, extending I-model with an additive genomic effect, and (iii) “I + G_A_ + G_AA_-model”, extending I + G_A_-model with an additive-by-additive genomic effects. The I + G_A_-model and I + G_A_ + G_AA_-model were based on the Natural and Orthogonal Interactions Approach (NOIA) parametrization. The I + G_A_ + G_AA_-model failed to achieve orthogonal partition of genetic variances, as revealed by a change in estimated additive variance of I + G_A_-model when epistasis was included in the I + G_A_ + G_AA_-model. The PA was studied using leave-one-line-out and leave-one-breeding-cycle-out cross-validations. The I + G_A_ + G_AA_-model increased PA significantly (16.5%) compared to the I + G_A_-model in leave-one-line-out cross-validation. However, the improvement due to including epistasis was not observed in leave-one-breeding-cycle-out cross-validation. We conclude that epistatic models can be useful to enhance predictions of total genetic merit. However, even though we used the NOIA parameterization, the variance partition into orthogonal genetic effects was not possible.

**Supplementary Information:**

The online version contains supplementary material available at 10.1007/s00122-021-04009-4.

## Introduction

Genomic selection (GS, Meuwissen et al. 2001) methods based on whole-genome prediction (WGP) have been successfully applied for a variety of quantitative traits of agronomic importance in animals and plants (Poland et al. 2012; Gianola and Rosa 2015; Crossa et al. 2017; Kristensen et al. 2019).

In quantitative genetics, a distinction is made between the genomic estimated breeding value (GEBVs, estimated additive genetic effects) and the total genetic value (estimated additive plus non-additive genetic effects). Traditionally, wheat breeders have based the selection of lines on phenotypic selection, which can be seen as a measure of total genetic value. The better performance of GS over phenotypic selection (Crossa et al. 2011; Michel et al. 2017; Tessema et al. 2020) has led many wheat breeding programs to implement GS, and base the selection of lines on the prediction of GEBVs, which in general are used to select both breeding lines and commercial varieties. However, the non-additive genetic effects can play a relevant role in the determination of complex traits such as grain yield (Carlborg and Haley 2004; Mackay 2014). Separating additive and non-additive genetic effects can be favorable if it contributes to a more accurate estimate of both additive and total genetic merit. In this context, treating additive and non-additive effects separately can result in an improved strategy of selection, allowing to select crossing parents based exclusively on the additive effect, and develop commercial varieties, based on both additive plus non-additive effects.

The non-additive genetic effect can be defined by their “biological” meaning, referred to the variations due to gene action, or as defined by Fisher (1918), by their “statistical” meaning, referred to deviations from additivity in a statistical model. The non-additive genetic effects are classified into epistasis and dominance. Epistasis is defined as the interaction between alleles at different loci, and it can be divided into pairwise classes: (i) additive-by-additive, (ii) additive-by-dominance and (iii) dominance-by-dominance, and into higher-order epistatic classes involving more than two loci. In wheat breeding, commercial cultivars are commonly developed by several generations of selfing to create inbred lines. Due to the high homozygosity of inbred lines obtained by seed multiplication via selfing, the epistatic interactions are fixed in cultivars and can be kept for future generations used in commercial production.

Modelling additive-by-additive effects in genomic prediction (GP) can be restrictive due to the high computational load caused by the high number of interactions between markers if all possible interactions are considered. Under the assumption of quantitative trait loci (QTL) effects coming from the same normal distribution, a mathematically equivalent alternative to model epistasis, and reduce the computational load, is to use models including genomic relationship matrices as covariance structures for individuals. Several authors have proposed to extend the genomic best linear unbiased prediction (G-BLUP) model (Habier et al. 2007; VanRaden 2008) by adding non-additive terms (extended best linear unbiased prediction, EG-BLUP). The term “EG-BLUP” refers in the literature to a model with multiple types of genetics effects (additive, dominance, epistatic), in which the coding of the marker matrix to calculate the relationship matrices can be flexible (Su et al. 2012; Xu 2013; Jiang and Reif 2015; Martini et al. 2016). Henderson (1985) proposed to use the Hadamard product of the pedigree-based additive relationship matrix with itself to approximate the additive-by-additive epistatic matrix. Henderson’s approach was later implemented in the genomic framework by Su et al. (2012), where the Hadamard product of the additive genomic relationship matrix was used to build the additive-by-additive matrix. The resulting marker-based relationship matrix captures deviations due to additive-by-additive interactions plus dominance when it is present (Martini et al. 2016). Marker-based epistatic relationship matrices are also proposed to estimate the additive-by-additive interactions without including the dominance effect (Xu 2013; Jiang and Reif 2015; Martini et al. 2016). Recently, Vitezica et al. (2017) proposed to use the natural and orthogonal interactions (NOIA) approach (Alvarez-Castro and Carlborg 2007) to model non-additive genetic effects in GP. However, as recently reported by Joshi et al. (2020), the EG-BLUP and the NOIA are equivalents if the marker coding for the EG-BLUP follows VanRaden (2008) and only additive and additive-by-additive epistatic effects are included in the models.

The dominance genetic effect has also been investigated in GS for wheat breeding. Dominance is defined as the effects of allelic interaction within loci (Fisher 1918), and it has been particularly relevant for the heterotic effect in hybrid wheat populations (Zhao et al. 2015; Jiang et al. 2017). Jiang et al. (2017) found a heterotic effect for grain yield in a hybrid population of winter wheat derived from crosses among diverse elite parents. In their study, the hybrids outperformed the mid-parents by 10% on average. The relevance of accounting for dominance in prediction models has also been investigated in simulation studies, reporting an increase in the prediction accuracy for populations presenting a dominance effect when dominance was accounted for in prediction models (Wellmann and Bennewitz 2012). However, for inbred wheat lines, the dominance effects are very low to negligible due to their reduced heterozygosity, and the epistasis is, therefore, the only relevant non-additive genetic effect.

The lack of independence between loci, and having linked markers instead of causative mutations may affect the orthogonal partition of genetic effects into independent statistical components and lead to problems in the estimation of genetic variances (Zeng et al. 2005; Wang and Zeng 2006; Hill and Mäki‐Tanila 2015; Vitezica et al. 2017). The lack of orthogonality between genetic effects can be evidenced by estimates that are affected when an additional genetic term is included in the model (Papoulis and Pillai 2002; Vitezica et al. 2017; Joshi et al. 2020). Nevertheless, several authors have reported that including epistasis in genetic models can be useful to enhance prediction and selection (Hu et al. 2011; He et al. 2016; Martini et al. 2017). On the other hand, different results have been reported by other authors, Jarquín et al. (2014) found that including epistasis did not improve PA, and Lorenzana and Bernardo (2009) even found a negative effect of including epistasis in PA.

In this study, we use a large set of winter wheat breeding lines, phenotyped for grain yield in multiple environments and in multiple years. Our study had two specific objectives:

(i) To evaluate the performance of models including additive and additive-by-additive epistatic effects for variance component estimation for grain yield.
(ii) To investigate the predictive ability (PA) of such models for prediction of advanced breeding lines.

## Materials and methods

### Experimental data

The plant material consisted of 2060 sixth-generation (F_6_) winter wheat lines (*T. aestivum* L.) developed by the breeding company Nordic Seed A/S. The data were collected from seven breeding cycles from 2013 to 2019, each including around 330 lines evaluated in three locations in Denmark (DK): Odder (Central DK), Holeby (South DK) and Skive (North DK). The F_6_ lines of each breeding cycle originated from approximately 60 parental line-crosses, followed by five generations of selfing, including creating single seed descent (SSD) lines in generation F_4_. The breeding cycles from 2013 to 2016 were evaluated in two consecutive years (cycle 1: 2013–2014, cycle 2: 2014–2015, cycle 3: 2015–2016, cycle 4: 2016–2017), and the cycles coming from 2017 to 2019 were evaluated in one year only (cycle 5: 2017, cycle 6: 2018, cycle 7: 2019). The field trials consisted of 15 blocks of 46 line plots of 8.25 m^2^ per year × location combination. Each block had two replicates of 21 F_6_ lines and two checks randomly assigned. The experimental conditions within the year × location subsets were homogeneous for the trials (e.g., sowing time, application of treatments, assessment time). The quantitative trait analyzed in this study was the yield measured as kg per plot (8.25 m^2^).

### Genotyping

DNA extractions from the plant material were based on a modified CTAB method (Rogers and Bendich 1985). The genotyping was carried out using a 15 K Illumina Infinium iSelect HD Custom Genotyping BeadChip technology (Wang et al. 2014). For the quality control, the SNPs with minor allele frequency (MAF) lower than 5% and with a call rate < 0.90 were removed. Missing genotypes were imputed with mean value (∼1.3% of missing values imputed). In total, 10,688 SNPs passed the quality control.

### Statistical models

This study compared three different models. Firstly, a baseline mixed model without genomic information (I-model, Eq. 1), including fixed and random effects, was used as the starting point for the construction of the other models (Cericola et al. 2017; Tsai et al. 2020). Secondly, the I + G_A_-model (Eq. 2) was used to extend the I-model with an additive genomic effect according to the NOIA parametrization proposed by Alvarez-Castro and Carlborg (2007) and later extended to GP by Vitezica et al. (2017). Third, the I + G_A_ + G_AA_-model (Eq. 4) was used to extend the I + G_A_-model by adding a pairwise additive-by-additive epistatic terms according to the NOIA parametrization.

#### *I-model(Baseline)*

The baseline model (Eq. 1) was developed considering the main sources of variability affecting the experimental data and included them as fixed or random effects, and we referred to as “I-model” hereinafter since it uses an identity covariance matrix for the line effects. A similar model has also been presented in earlier studies working with a set of data from Nordic Seed A/S (Cericola et al. 2017; Tsai et al. 2020). The I-model was defined as:

1 $${\varvec{y}} = {\varvec{Xb}} + {\varvec{Z}}_{1} {\varvec{l}} + {\varvec{Z}}_{2} {\varvec{f}} + \mathop \sum \limits_{i = 1}^{9} {\varvec{Z}}_{{{\varvec{i}} + 2}} {\varvec{s}} + {\varvec{e}}$$

where ${\varvec{y}}$ is the vector of observed phenotypes; ${\mathbf{X}}$ is the design matrix for fixed effects; ${\varvec{b}}$ is the vector of fixed trial effects nested within year, location and breeding cycle; ${\mathbf{Z}}_{1}$ and ${\mathbf{Z}}_{2}$ are design matrices of random effects; ${\varvec{l}}$ is a vector of line effect with $\user2{l }\sim N\left( {0,{\varvec{I}}\sigma_{l}^{2} } \right)$, where ${\varvec{I}}$ is an identity matrix and $\sigma_{l}^{2}$ is the variance due to uncorrelated line effects; ${\varvec{f}}$ is a vector of line by environment interaction (lines-by-year-location) with $\user2{f }\sim N\left( {0,{\varvec{I}}\sigma_{f}^{2} } \right)$, where $\sigma_{f}^{2}$ is the variance due to uncorrelated line by environment effects; ${\varvec{s}}$ is a vector of spatial effect with $\user2{s }\sim N\left( {0,{\varvec{I}}\sigma_{s}^{2} } \right)$, where $\sigma_{s}^{2}$ is the spatial effect variance. The spatial effect contains the X and Y coordinate of the target plot and the eight surrounding plots (*n* = 9), for plots located in the border, virtual plots were added to guarantee all plots have *n* = 9 in order to account for border effects (Supplementary material Fig. 1S). Therefore, the spatial effect on an individual plot is the sum of effects with the square centered on the plot itself plus the effects of eight surrounding plots with a square centered on those plots; ${\varvec{e}}$ is a vector of random residuals with $\user2{e }\sim N\left( {0,{\varvec{I}}\sigma_{e}^{2} } \right)$, where $\sigma_{e}^{2}$ is the residual variance. All random effects were assumed to be independent.

Note that the genetic term in the I-model is miss-specified since the model assumes all lines to be unrelated. Therefore, it may lead to a biased estimation of the total genetic variance.

#### *I* + *GA-model*

The “I + G_A_-model” (Eq. 2) was the second model used, and it includes an additive genomic relationship matrix based on the NOIA parametrization as covariance structure to define the additive genetic effects. The I + G_A_-model was defined as:

2 $${\varvec{y}} = {\varvec{Xb}} + {\varvec{Z}}_{1} {\varvec{l}} + {\varvec{Z}}_{3} {\varvec{g}} + {\varvec{Z}}_{2} {\varvec{f}} + \mathop \sum \limits_{i = 1}^{9} {\varvec{Z}}_{{{\varvec{i}} + 3}} {\varvec{s}} + {\varvec{e}}$$

where ${\mathbf{X}}$, ${\mathbf{Z}}_{{\mathbf{n}}}$, ${\varvec{b}}$, ${\varvec{l}}$, ${\varvec{f}}$, ${\varvec{s}}$, and ${\varvec{e}}$ are the same as described in the I-model (Eq. 1); ${\varvec{g}}$ is a vector of additive genomic breeding values with $\user2{g }\sim N\left( {0,{\mathbf{G}}_{{{\mathbf{NOIA}}}} \sigma_{g}^{2} } \right)$, where $\sigma_{g}^{2}$ is the genomic additive genetic variance and ${\mathbf{G}}_{{{\mathbf{NOIA}}}}$ is a genomic relationship matrix constructed based on Vitezica et al. (2017):

3 $${\varvec{G}}_{{{\varvec{NOIA}}}} = \frac{{{\varvec{H}}_{{\varvec{a}}} {\varvec{H}}_{{\varvec{a}}}^{\prime } }}{{tr\left( {{\varvec{H}}_{{\varvec{a}}} {\varvec{H}}_{{\varvec{a}}}^{\prime } } \right)/n}}$$

where ${\varvec{H}}_{{\varvec{a}}}$ is an *n* rows (number of lines) x *m* columns (number of markers) matrix containing the additive coefficients as:

$${\varvec{H}}_{{\varvec{a}}} = \left( {\begin{array}{*{20}c} {{\varvec{h}}_{{{\varvec{a}}_{{\varvec{i}}} }} } \\ {\begin{array}{*{20}c} . \\ . \\ . \\ {{\varvec{h}}_{{{\varvec{a}}_{{\varvec{n}}} }} } \\ \end{array} } \\ \end{array} } \right)$$

and ${\varvec{h}}_{{{\varvec{a}}_{{\varvec{i}}} }}$ is a row vector for the *i*th individual with *m* columns. For individual 1 with marker *j* = 1,.., *m*, the element ${\varvec{h}}_{{{\varvec{a}}_{1} {\varvec{j}}}}$ is equal to:

$${\varvec{h}}_{{{\varvec{a}}_{1} {\varvec{j}}}} = \left\{ {\begin{array}{*{20}c} {\begin{array}{*{20}c} { - \left( {p_{Aa} - 2p_{aa} } \right)} \\ { - \left( {1 - p_{Aa} - 2p_{aa} } \right)} \\ \end{array} } \\ { - \left( {2 - p_{Aa} - 2p_{aa} } \right)} \\ \end{array} } \right.{\text{for genotypes}}\;\left\{ {\begin{array}{*{20}c} {\begin{array}{*{20}c} {AA} \\ {Aa} \\ \end{array} } \\ {aa} \\ \end{array} } \right.$$

where $p_{Aa}$ and $p_{aa}$ are the genotypic frequencies for the genotypes *Aa* and *aa* in locus *A*. The term $tr\left( {{\varvec{H}}_{{\varvec{a}}} {\varvec{H}}_{{\varvec{a}}}^{\prime } } \right)/n$ is the trace for the ${\varvec{H}}_{{\varvec{a}}} {\varvec{H}}_{{\varvec{a}}}^{\prime }$ matrix, which standardize ${\varvec{G}}_{{{\varvec{NOIA}}}}$ to a variance equal to one.

#### *I* + *GA* + *GAA-model*

Our last model, extend I + G_A_-model by including an additive-by-additive epistatic term using a genomic relationship matrix based on NOIA parametrization (Alvarez-Castro and Carlborg 2007; Vitezica et al. 2017) as covariance structures. The I + G_A_ + G_AA_-model was defined as:

4 $${\varvec{y}} = {\varvec{Xb}} + {\varvec{Z}}_{1} {\varvec{l}} + {\varvec{Z}}_{3} {\varvec{g}} + {\varvec{Z}}_{4} {\varvec{h}} + {\varvec{Z}}_{2} {\varvec{f}} + \mathop \sum \limits_{i = 1}^{9} {\varvec{Z}}_{{{\varvec{i}} + 4}} {\varvec{s}} + {\varvec{e}}$$

where ${\mathbf{X}}$, ${\mathbf{Z}}_{{\mathbf{n}}}$, ${\varvec{b}}$, ${\varvec{l}}$, ${\varvec{f}}$, ${\varvec{s}}$, ${\varvec{g}}$, and ${\varvec{e}}$ are the same as described in the I-model (Eq. 1) and I + G_A_-model (Eq. 2); ${\varvec{h}}$ is a vector of epistatic genomic values for the lines with $\user2{h }\sim N\left( {0,{\varvec{H}}_{{{\varvec{NOIA}}}} \sigma_{h}^{2} } \right)$, where $\sigma_{h}^{2}$ is the genomic epistatic variance and ${\varvec{H}}_{{{\varvec{NOIA}}}}$ is the epistatic relationship matrix constructed based on Vitezica et al. (2017):

5 $${\varvec{H}}_{{{\varvec{NOIA}}}} = \frac{{{\varvec{G}}_{{{\varvec{NOIA}}}} \odot {\varvec{G}}_{{{\varvec{NOIA}}}} }}{{tr\left( {{\varvec{G}}_{{{\varvec{NOIA}}}} \odot G_{NOIA} } \right)/n}}$$

where the $\odot$ operator represents the Hadamard product between matrices, and the term $tr\left( {{\varvec{G}}_{{{\varvec{NOIA}}}} \odot {\varvec{G}}_{{{\varvec{NOIA}}}} } \right)/n$ is the trace for the ${\varvec{G}}_{{{\varvec{NOIA}}}} \odot {\varvec{G}}_{{{\varvec{NOIA}}}}$ matrix, which standardize ${\varvec{H}}_{{{\varvec{NOIA}}}}$ to a variance one.

As explained in the introduction, the model following NOIA parametrization is equivalent to a EG-BLUP model following VanRaden (2008) coding since in the current scenario, only additive and additive-by-additive effects are considered in the models, and no dominance effect is present in the population (Joshi et al. 2020).

### Variance components and heritability

The estimation of variance components (VC) was performed using the Average Information Restricted Maximum Likelihood (AI-REML) algorithm in the DMU software (Madsen and Jensen 2013). The phenotypic variance of the plot ($\sigma_{P}^{2}$) for the I + G_A_-model (Eq. 2) was calculated as:

6 $$\hat{\sigma }_{P}^{2} = \hat{\sigma }_{l}^{2} + \hat{\sigma }_{g}^{2} + \hat{\sigma }_{f}^{2} + 9\hat{\sigma }_{s}^{2} + \hat{\sigma }_{e}^{2}$$

where $\hat{\sigma }_{l}^{2}$ is the estimated variance of the line that cannot be attributed to the markers; $\hat{\sigma }_{g}^{2}$ is the genomic estimated additive variance; $\hat{\sigma }_{f}^{2}$ is the line by environmental estimated variance; $9\hat{\sigma }_{s}^{2}$ is the estimated spatial variance for an individual plot ($\hat{\sigma }_{s}^{2}$) multiplied by nine, which is the total number of plots considered as random effect for each observation; $\hat{\sigma }_{e}^{2}$ is the estimated variance of residuals. Narrow-sense (Eq. 7) and broad-sense (Eq. 8) plot heritabilities for the I + G_A_-model (Eq. 2) were estimated as:

7 $$\hat{h}^{2} = \hat{\sigma }_{g}^{2} /\hat{\sigma }_{P}^{2}$$

8 $$\hat{H}^{2} = \left( {\hat{\sigma }_{l}^{2} + \hat{\sigma }_{g}^{2} } \right)/\hat{\sigma }_{P}^{2}$$

Additionally, for the I + G_A_ + G_AA_-model (Eq. 4), the estimated epistatic variance ($\hat{\sigma }_{h}^{2}$) was considered in the calculation of broad-sense heritability and total phenotypic variance ($\hat{\sigma }_{P}^{2}$) for this model. For the I-model, only the broad-sense heritability was calculated. The total genetic variance for each model ($\hat{\sigma }_{G}^{2}$) was defined as $\hat{\sigma }_{l}^{2}$ for the I-model, $\hat{\sigma }_{l}^{2}$ + $\hat{\sigma }_{g}^{2}$ for I + G_A_-model, and $\hat{\sigma }_{l}^{2}$ + $\hat{\sigma }_{g}^{2}$ + $\hat{\sigma }_{h}^{2}$ for the I + G_A_ + G_AA_-model.

### Cross-validation schemes and model validation

The PA ($r_{{\widehat{g,}p}}$) of the models was evaluated using two cross-validation (CV) schemes: (i) leave-one-line-out (LOO), and (ii) leave-one-breeding-cycle-out (LSO) CVs. The LOO CV scheme was used to get the PA with the largest reference population possible and investigate the potential performance of the genetic models on PA. The LOO strategy was performed by masking the phenotype of a single line and using the remaining lines to predict the GEBV and the Genomic Estimated Epistatic Value (GEEV) of the masked line. This methodology was repeated n-times (*n* = no. of lines = 2060) until all lines were predicted. The LSO CV was used to measure the PA of genetic models in conditions closer to those observed in wheat breeding programs. For LSO the phenotypes from a breeding cycle were masked, and the information from the remaining breeding cycles was used to predict the genetic values. This process was repeated n-times (*n* = no. of breeding cycles = 7) until all breeding cycles were predicted. The PA was calculated as the Pearson correlation between the vector of all predictions and the lines averages after correcting for the fixed effects. The predicted values were the additive predicted values (predicted GEBVs) for the I + G_A_-model and I + G_A_ + G_AA_-model, and the additive (predicted GEBVs) plus epistatic (predicted GEEVs) values for the I + G_A_ + G_AA_-model. The fixed effects were estimated in a model using the complete phenotypic information. The line averages were computed first subtracting the estimates of the fixed effects from each plot observation and then averaging the values of the lines without fixed effect across year-locations and repetitions. To contrast the PA for models in the LOO CV scenario, an ordinary nonparametric bootstrap with replacement based on a sample size equal to *n* = 2060 (full sample size), and 10,000 replicates was performed. In each bootstrap replication, the PA was recorded until reaching 10,000 bootstrap-based PAs, and the standard error of PAs was obtained. The bootstrap procedure was performed for I + G_A_-model and I + G_A_ + G_AA_-model, and a two-tailed paired *t*-test was used to contrast the bootstrap PAs from both models (significance threshold set at 0.01). The relative difference (RD) in PA between prediction for the additive genetic effect using I + G_A_-model (GEBVs) and total genetic effect using I + G_A_ + G_AA_-model (GEBVs + GEEVs) was estimated as: $RD = \frac{{I + G_{A} + G_{AA} \hbox{-}modelr_{{\widehat{g,}p}} - I + G_{A} \hbox{-}modelr_{{\widehat{g,}p}} }}{{ I + G_{A} \hbox{-}modelr_{{\widehat{g,}p}} }}$. The maximum potential PA was calculated for the I + G_A_-model and for the GEBVs of the I + G_A_ + G_AA_-model as: $\sqrt {{\text{n}}h^{2} /\left( {1 + \left( {{\text{n}} - 1} \right)h^{2} } \right)}$, where n is the average number of lines repetitions, and for the GEBVs + GEEVs of the I + G_A_ + G_AA_-model using the same equation but with the proportion of total variance explained by additive plus epistatic effects instead of $h^{2}$.

The statistics for bias ($\mu_{wp}$) and variance inflation ($b_{w,p}$) in the predicted genetic values were estimated according to the LR method (Legarra and Reverter 2018). The $\mu_{wp}$ was calculated as $\mu_{wp} = E\left( {\overline{{\widehat{{{\text{u}}_{p} }}}} - \overline{{\widehat{{{\text{u}}_{w} }}}} } \right)$; where $\overline{{\widehat{{{\text{u}}_{p} }}}}$ represents the mean of the genomic estimated values with “partial” (subscript *p*) information (predictions for all genotypes from CVs when their own phenotypes were masked, e.g., 2060 “partial” dataset of one line and seven “partial” dataset of one breeding cycle were generated for LOO and LSO CVs, respectively), and $\overline{{\widehat{{{\text{u}}_{w} }}}}$ represents the mean of the genomic estimated values with “whole” (subscript *w*) information (estimations with complete phenotypic information for all genotypes). The statistics $\mu_{wp}$ has an expected value of 0 when the estimations are unbiased. The $b_{w,p}$ was calculated as the regression of estimated values obtained with whole information (subscript *w*) on the estimated with partial information (subscript *p*), $b_{w,p} = \frac{{cov\left( {\widehat{{{\text{u}}_{w} }} , \widehat{{{\text{u}}_{p} }}} \right)}}{{var\left( {\widehat{{{\text{u}}_{p} }}} \right)}}$. The statistic $b_{w,p}$ has an expectation $E\left( {b_{w,p} } \right) = 1$ when there is no under- or over-dispersion in the predictions. Additionally, the Pearson correlation was used to compare predictions between models, where the correlation between the estimated values with whole information for the I + G_A_-model and the I + G_A_ + G_AA_-model ($\rho_{{I + G_{A} \hbox{-}model_{GEBV} , I + G_{AA} + G_{AA} \hbox{-}model_{GEBV} }}$ and $\rho_{{I + G_{A} \hbox{-}model_{GEBV} , I + G_{A} + G_{AA} \hbox{-}model_{GEEV} }}$) was calculated.

## Results

### Phenotyping and genotyping

The descriptive statistics for grain yield are presented in Table 1. The average yield was 8.71 kg of grain for an 8.25 m^2^ plot, ranging from 3.85 to 12.35 kg/8.25 m^2^, and the coefficient of variation was 11.27% when using the simple SD of all observations.

**Table 1:** Descriptive statistics for the yield of F6 wheat breeding lines

Breeding cycle	No. of lines	No. of plots	Average (SD)*	Min. value*	Max. value*	Coefficient of variation (%)
1	325	4080	8.77 (0.76)	5.28	11.17	8.67
2	325	3408	8.66 (0.79)	3.85	11.00	9.10
3	245	2862	8.65 (1.16)	4.68	11.46	13.35
4	336	3360	8.28 (1.09)	4.99	11.65	13.19
5	159	954	9.14 (0.99)	6.10	11.33	11.03
6	358	1789	8.59 (0.46)	7.06	10.25	5.36
7	312	2491	9.35 (1.06)	6.04	12.36	12.40
Total	2060	18,525	8.71 (0.98)	3.85	12.35	11.27

*Units of measure: kg grain/8.25 m^2^; No.: number; SD: standard deviation; Min: Minimum; Max: Maximum

**Fig. 1:**
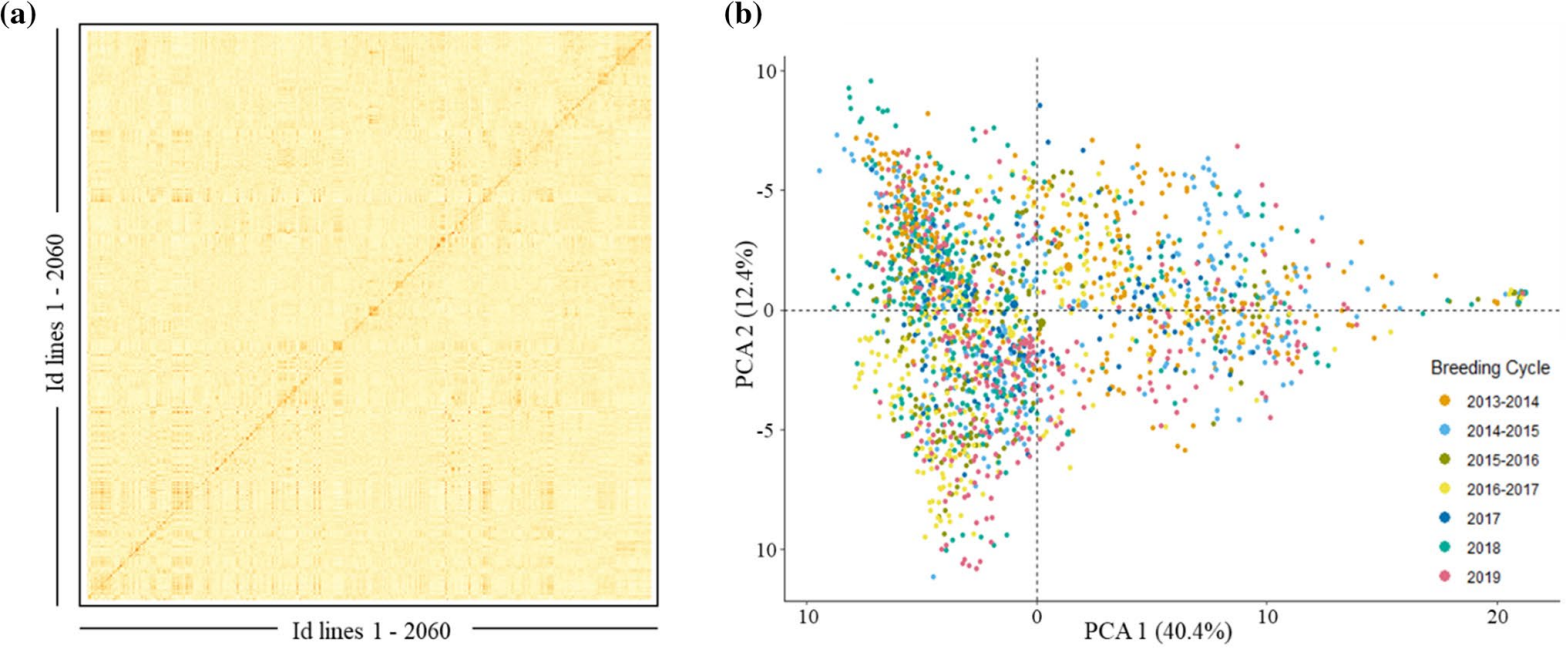
Genomic relationship between the 2060 F_6_ lines from 2013 to 2019 breeding cycles. **a** Heat map of G-matrix, red colors represents more related individuals and yellow colors less related. **b** Principal component analysis (PCA) of G-matrix. The colors of the PCA represent the different breeding cycles to which the lines correspond. The variances explained by PCA1 and PCA2 are 40.4 and 12.4%, respectively (color figure online)

A total of 10,688 SNPs passed the quality control filters and were used to build the genomic relationship matrices. According to the heat map and the principal component analysis of the G-matrix (Fig. 1), there was no clear separation of breeding cycles. However, there was a trend that lines coming from the first four breeding cycles were more separated by the first principal component from lines coming from last three breeding cycles. The first and second principal components together explained 52.8% of the total variance (40.4 and 12.4% of the variance for first and second principal component, respectively) showing that there are strong relationships between the lines included in the study. The observed level of heterozygosity of the lines had an average value of 2.70% as expected after five generations of selfing.

### Variance components and heritability

Three models differing in how the genetic components were treated (Eqs. 1, 2, and, 4) were used to estimate VC and the narrow-sense and broad-sense plot heritabilities (Table 2). The estimates for total phenotypic ($\hat{\sigma }_{P}^{2}$) and error variance ($\hat{\sigma }_{e}^{2}$) were similar for all models. The highest variance was attributed to the genotype-by-environment interaction, which explained around 40% of the total variability. The estimated total genetic variance ($\hat{\sigma }_{G}^{2}$) was largest when the I + G_A_-model (0.104) or I + G_A_ + G_AA_-model (0.098) were used, followed by the baseline model with the lowest value (0.089). The models using genomic information captured around 15% more $\hat{\sigma }_{G}^{2}$ compared to the baseline model.

**Table 2:** Estimation of variance component, narrow-sense, and broad-sense plot heritabilities

Models	${{\sigma}}_{{{{Line}}}}^{2}$	${{\sigma}}_{{{{Additive}}}}^{2}$	${{\sigma}}_{{{{Epistatic}}}}^{2}$	${{\sigma}}_{{{{Spatial}}}}^{2}$	${{\sigma}}_{{{{GxE}}}}^{2}$	${{\sigma}}_{{{{error}}}}^{2}$	*Plot heritabilities*	
							h^2^*	H^2^
I-model	0.089 (0.004)	–	–	0.043 (0.002)	0.131 (0.003)	0.057 (0.001)	–	0.28
I + G_A_-model	0.053 (0.004)	0.051 (0.007)	–	0.044 (0.002)	0.131 (0.003)	0.057 (0.001)	0.15	0.31
I + G_A_ + G_AA_-model	0.014 (0.005)	0.020 (0.006)	0.064 (0.008)	0.044 (0.002)	0.131 (0.003)	0.057 (0.001)	–	0.30

*The narrow-sense heritability (h^2^) was estimated only for the I + G_A_-model, due to the lack of orthogonality of genetic components, h^2^ was not representative for the I + G_A_ + G_AA_-model. $\sigma_{Line}^{2}$: variance not captured by markers; $\sigma_{Additive}^{2}$: additive variance; $\sigma_{Epistatic}^{2}$: epistatic variance; $\sigma_{Spatial}^{2}$: spatial variance; $\sigma_{LxE}^{2}$: line by environment interaction variance; $\sigma_{error}^{2}$: error variance; H^2^: broad-sense heritability. The values between parentheses are the standard errors (SE) of the estimates

**Fig. 2:**
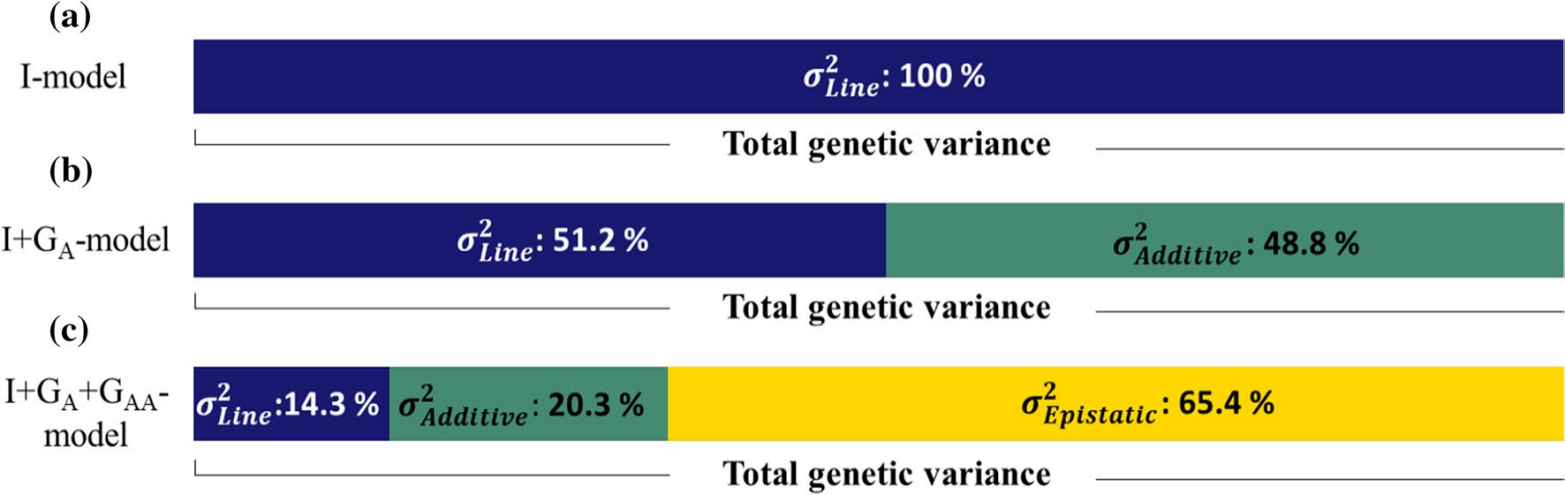
Percentage of genetic variances (blue: $\sigma_{Line}^{2}$, green: $\sigma_{Additive}^{2}$, yellow:$\sigma_{Epistatic}^{2}$) captured by the different models. **a** Genetic variance estimated for the I-model; the variance of the line ($\sigma_{Line}^{2}$) represents a combination of additive plus non-additive variances. **b** Genetic variances estimated for I + G_A_-model; $\sigma_{Line}^{2}$ represents the non-additive variance plus the additive variance not captured by SNPs, $\sigma_{Additive}^{2}$ represents the additive variance captured by SNPs. **c** The genetic variances estimated for the I + G_A_ + G_AA_-model. Under an orthogonal partition of variances into genetic components, $\sigma_{Line}^{2}$ is expected to reflect the additive and non-additive variance that was not captured by SNPs, and $\sigma_{Additive}^{2}$ and, $\sigma_{Epistatic}^{2}$ are expected to represent the additive and the pairwise additive-by-additive epistatic variance captured by SNPs, respectively (color figure online)

The partition of total genetic variance $\hat{\sigma }_{G}^{2}$ estimated by the different models is shown in Fig. 2. For the I + G_A_-model, the estimated additive variance ($\hat{\sigma }_{g}^{2}$) was approximately half of the total genetic variance $\hat{\sigma }_{G}^{2}$ (48.8%). The partition of the estimated variances for the I + G_A_ + G_AA_-model changed considerably compared to the I + G_A_-model. The estimate of additive genetic variances ($\hat{\sigma }_{g}^{2}$ for I + G_A_ + G_AA_-model) was reduced to approximately 20%, and the estimated epistatic variances ($\hat{\sigma }_{h}^{2}$) represented 65.4% of the total genetic variance $\hat{\sigma }_{G}^{2}$. Note that the inclusion of an epistatic term captured much of what had previously been part of the estimated line and additive variances in the I + G_A_-model. The reduction in the additive variance when the epistatic effect is included in the model can be seen as a signal of lack of orthogonality between the additive and additive-by-additive genetic effects.

The H^2^ estimate was slightly different for the genomic models I + G_A_-model (0.31) and I + G_A_ + G_AA_-model (0.30), and in both cases, it was higher than the I-model (0.28), which did not include genomic information. The narrow-sense heritability estimated (h^2^) for the I + G_A_-model on the plot level had a value of 0.15. For the I + G_A_ + G_AA_-model, h^2^ was not estimated because the estimation of additive variance may be especially affected due to the lack of orthogonality among genetic effects when the epistatic genetic effect is considered in the model.

### Genomic prediction

**Fig. 3:**
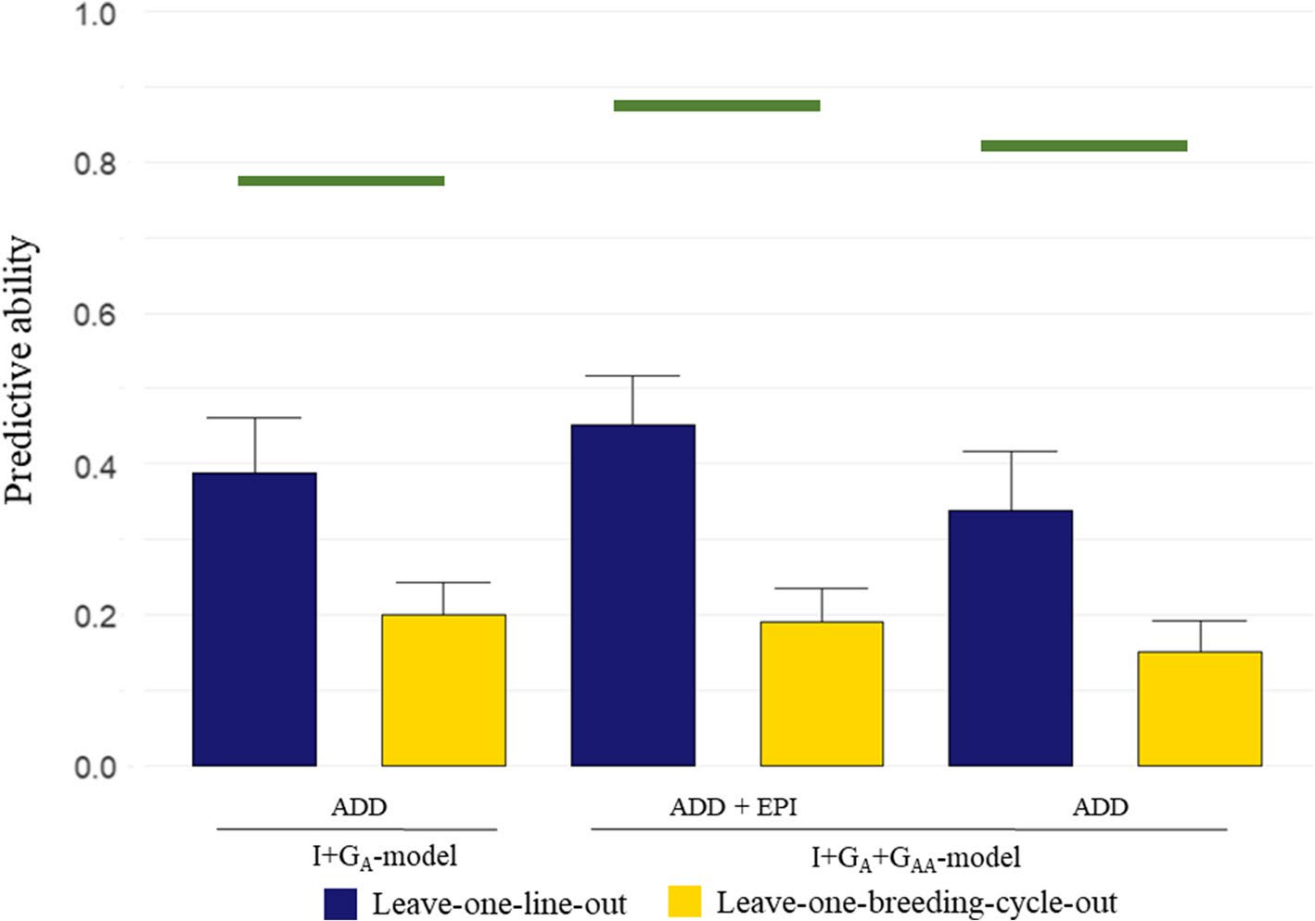
Barplot of predictive abilities for I + G_A_-model and I + G_A_ + G_AA_-model in leave-one-line-out (LOO) and leave-one-breeding-cycle-out (LSO) cross-validations based on bootstrap distribution, *r* = 10,000. ADD: predicted additive values (GEBVs), EPI: predicted epistatic values (GEEVs), ADD + EPI: sum of ADD and EPI. Green lines are the theoretical maximum predictive ability (PA). The maximum PA for the I + G_A_-model and for the ADD of I + G_A_ + G_AA_-model were calculated as: $\sqrt {{\text{n}}h^{2} /\left( {1 + \left( {{\text{n}} - 1} \right)h^{2} } \right)}$, where *n* is the average number of lines repetitions; the maximum PA for ADD + EPI of I + G_A_ + G_AA_-model was calculated using the proportion of total variance explained by additive plus epistatic effects instead of $h^{2}$ (color figure online)

The PA between the lines averages after correcting for fixed effects and the predicted genetic values (${\varvec{r}}_{{\widehat{{{\varvec{g}},}}{\varvec{p}}}}$) was evaluated for the proposed models using LOO and LSO CV schemes (Fig. 3). The I-model was not included in this section because such a model has no PA in CVs due to the model assumptions of independence between lines.

In the LOO CV, the highest PA was observed for prediction of total genetic merit (additive plus epistatic genetic effects), combining the predictions for the additive effect (GEBVs) plus the predictions for the epistatic effect (GEEVs) from the I + G_A_ + G_AA_-model (PA = 0.45). The theoretical maximum PA was also the highest for the I + G_A_ + G_AA_-model when additive plus epistatic predictions were combined (green bars in Fig. 3). The PA of the I + G_A_ + G_AA_-model for total genetic merit (PA = 0.45) was contrasted to the PA of the I + G_A_-model for the additive effect (PA = 0.39), and it was significantly different in a two-tailed paired *t*-test (significance threshold set at 0.01), showing an increase of 16.5% in PA for the I + G_A_ + G_AA_-model. For the LSO CV scheme, the highest PA between predicted genetic values and corrected phenotypes was reached when the GEBVs from the I + G_A_-model were used, PA = 0.20, while the PA using the GEBVs plus GEEVs from I + G_A_ + G_AA_-model was 0.19. Nevertheless, the difference in PA between models for LSO CV was not significant in a two-tailed paired *t* test (significance threshold set at 0.01).

### Model validation

**Fig. 4:**
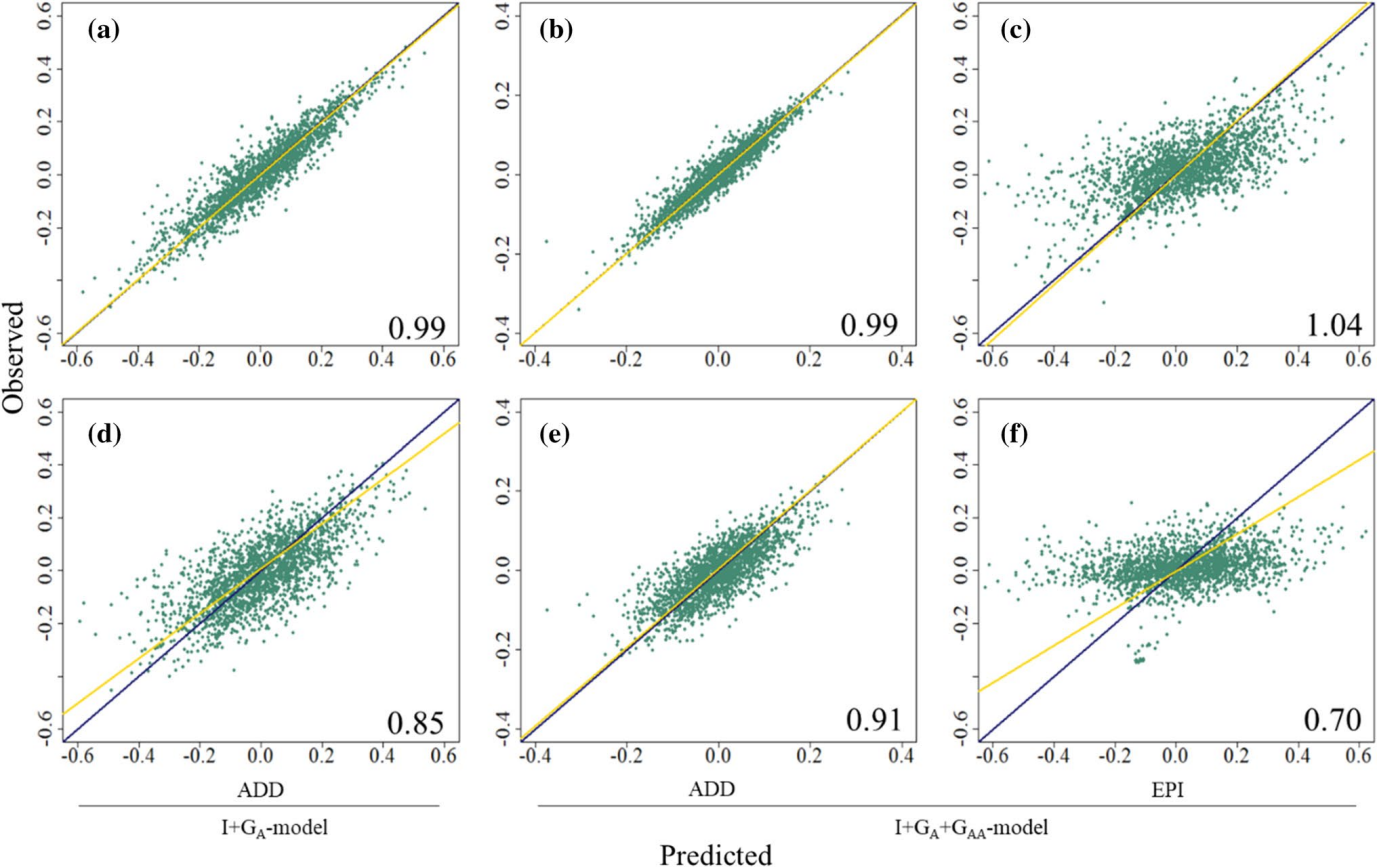
Slope of regression ($b_{w,p}$) among observed and predicted genetic values for I + G_A_-model and I + G_A_ + G_AA_-model in leave-one-line-out (LOO) cross-validation (**a**–**c**) and leave-one-breeding-cycle-out (LSO) cross-validation (**d**–**f**). The yellow lines represent the line for regression of observed on predicted genetic values. The blue lines represent a reference regression line with intercept 0 and slope 1. ADD: predicted additive values (GEBVs), EPI: predicted epistatic values (GEEVs). The numeric values into each plot represent the coefficient of regression ($b_{w,p}$) for each case (color figure online)

The regression coefficient ($b_{w,p}$), used as a test of variance inflation in the predicted genetic effects, was measured as the slope of the regression between observed and predicted values (Fig. 4). In the LOO CV, the $b_{w,p}$ did not present significant under- or over-dispersion since it had values around 1 for both models (Fig. 4a–c). The GEBVs from I + G_A_-model and I + G_A_ + G_AA_-model had a $b_{w,p}$ value of 0.99, while the GEEVs presented a value of 1.04. The $b_{w,p}$ was also estimated for the combination of I + G_A_ + G_AA_-model predictions (GEBVs + GEEVs, data not displayed in the plot), which presented an intermediate value of 1.02. In the LSO CV, the $b_{w,p}$ statistic indicates over-dispersion (inflation) for predicted values since it had values below 1 (Fig. 4d–f). The GEBVs from I + G_A_-model and I + G_A_ + G_AA_-model had $b_{w,p}$ values of 0.85 and 0.91, respectively, while the GEEVs from the I + G_A_ + G_AA_-model had a lower $b_{w,p}$ value of 0.70. The $b_{w,p}$ for the combination of I + G_A_ + G_AA_-model predictions (GEBVs + GEEVs, data not displayed in the plot) presented an intermediate value of 0.78.

The bias in prediction of genetic values ($\mu_{wp}$) was analyzed following the LR method (Table 3). For both LOO and LSO CVs, all the predictions showed a $\mu_{wp}$ close to 0 for I + G_A_-model and I + G_A_ + G_AA_-model, which reflects unbiased estimation for all cases.

**Table 3:** Estimated bias ($\mu_{wp}$) for predictions of I + G_A_-model and I + G_A_ + G_AA_-model

Model	Model	Genetic effect	Bias (${{\mu}}_{{{{wp}}}}$)
Leave-one-line-out cross-validation	I + G_A_-model	ADD	0.0005
	I + G_A_ + G_AA_-model	ADD	0.0004
		EPI	0.0013
Leave-one-breeding-cycle-out cross-validation	I + G_A_-model	ADD	− 0.0108
	I + G_A_ + G_AA_-model	ADD	− 0.0045
		EPI	− 0.0123

ADD: predicted additive values (GEBV), EPI: predicted epistatic values (GEEV)

### Correlation between G-BLUP and NOIA predictions

The additive and epistatic predictions using complete phenotypic information for the I + G_A_-model and the I + G_A_ + G_AA_-model were compared using Pearson’s correlation ($\rho_{{G\hbox{-}BLUP_{w} NOIA_{w} }}$). The correlation for GEBVs between I + G_A_-model and I + G_A_ + G_AA_-model had a high value of 0.94, while the correlation between GEBVs from I + G_A_-model and GEEVs from I + G_A_ + G_AA_-model had a lower value of 0.65. It was also reflected in the ranking of the best lines for the different genetic effects, where 7 of the 10 lines with highest GEBVs were common for predictions of I + G_A_-model and I + G_A_ + G_AA_-model, but when GEBV from I + G_A_-model and GEEV from I + G_A_ + G_AA_-model were compared, only 3 of 10 lines were common.

## Discussion

In this study, we investigated the performance of the NOIA parametrization (I + G_A_-model and I + G_A_ + G_AA_-model) in the estimation of VC for a set of advanced wheat breeding lines from the commercial breeding company Nordic Seed A/S. The I + G_A_ + G_AA_-model was not able to achieve an orthogonal estimation of genetic variance components as revealed by the difference of the estimated additive variance between I + G_A_-model and I + G_A_ + G_AA_-model. We also investigated the PA for the developed models in two CVs schemes: (i) leave-one-line-out and (ii) leave-one-breeding-cycle-out. We observed a significant increase of 16.5% (*P-value* < 0.01) in the PA for the LOO CV when I + G_A_ + G_AA_-model was used to predict total genetic merit compared to I + G_A_-model predictions. However, the improvement for including epistasis was not observed in the LSO CV, where no significant differences between PA from I + G_A_-model and I + G_A_ + G_AA_-model were observed.

### Variance components

The partition of genetic variance through the NOIA parametrization led to problems of non-orthogonality of genetic effects. The clearest signal of lack of orthogonality was observed in the difference of the estimated additive variance between I + G_A_-model and I + G_A_ + G_AA_-model. When the epistatic effect was present in the I + G_A_ + G_AA_-model, it caused a considerable reduction in the additive variance (58.4% of reduction) compared to the I + G_A_-model estimation. The non-orthogonal partition of genetic variances can most likely be caused as result of a mix between lack of independence of causal effect, lack of independence of markers (both influenced by LD), and for having linked markers instead of causative mutations (Wang and Zeng 2006; Hill and Mäki‐Tanila 2015; Vitezica et al. 2017). The lack of orthogonality of genetic effects can be also evidenced in the high negative correlation (−0.36) among additive and additive-by-additive epistatic variance component estimates for the I + G_A_ + G_AA_-model. For an orthogonal partition of variance into genetic components, the correlation between the variance component estimates is expected to be close to 0 (correlation of zero indicates independence between model effects). These results have also been consistent with the simulation study performed by Vitezica et al. (2017), where they tested the performance of the NOIA parametrization for an LD simulated population, and concluded that VC were wrongly estimated. In our study, negative correlations among genetic variance estimates were also observed between the line and additive effect for the I + G_A_-model (−0.44) and line and epistatic effects for the I + G_A_ + G_AA_-model (−0.74). These trends are expected since the line (*l*) effect in the I-model can be seen as a mix of additive and non-additive effects. Therefore, when the additive effect is included in the I + G_A_-model, it takes the proportion of additive variance explained by SNPs. Then, the line covariance of *l* in the I + G_A_-model can be interpreted as an estimate of remaining non-additive effects which can be partially captured by the epistatic effect in the I + G_A_ + G_AA_-model.

### Narrow and broad-sense heritability

The interpretation of the h^2^ is strongly related to the orthogonality of the estimated genetic variances. When additive and non-additive genetic effects are considered in genomic models, the lack of orthogonality affects the estimation of h^2^. Due to this issue, we analyzed h^2^ only for the I + G_A_-model, which does not include the definition of a genomic epistatic term. Note that in our study, we have approached the heritability calculations considering the line effect (${\varvec{l}}$) in the models. This approach was used in order to have control for the genetic factors (additive and non-additive) that are not captured by the markers in the genomic terms. The h^2^ estimated using the I + G_A_-model was 0.15, representing around half of the total genetic variation, and H^2^ was 0.31 to 0.30 for I + G_A_-model and I + G_A_ + G_AA_-model, respectively. The difference between h^2^ and H^2^ is given by non-additive variance and by remaining additive variance not captured by the markers (e.g., due to imperfect LD between markers and QTLs). The sizable difference between h^2^ and H^2^ may suggest a significant non-additive effect for wheat grain yield in the analyzed population, which also agrees with prior expert-knowledge from the breeding company.

### Genomic predictive ability

The PAs estimated as the correlation between the line averages after correcting for fixed effects and the predicted values of the I + G_A_-model and the I + G_A_ + G_AA_-model were estimated for the LOO and LSO CV schemes. Note that while the LOO CV is useful for model comparison and investigating the potential PA of genetic models, this scheme provides higher PAs than expected for breeding situations (Shao 1993; Kohavi 1995); conversely, the LSO CV better reflects the conditions in a breeding scenario where new lines must be predicted before phenotypes are obtained. In the LOO CV, the PA for I + G_A_ + G_AA_-model combining the predictions for the additive effect (GEBVs) plus the predictions for the epistatic effect (GEEVs) outperformed the I + G_A_-model PA using GEBVs with a significant (*P-value*: 0.01) increase of 16.5%. However, the improvement in PA for including epistasis was not observed when the LSO CV was used. The differences in the performance of models in the LOO and LSO CVs indicates a strong influence of relationships among individuals from the reference and validation population over the PA, as close relatives like full sibs are excluded in the LSO scenario. A possible explanation for the effect of genetic relationships on the performance of epistatic predictions could be related to the fact that the additive-by-additive effect is the result of a pairwise interaction, and it is more likely that the pairs involved in the interaction are present in close relatives as usually happen in the LOO CV but not in the LSO CV. Another factor that could be affecting the predictive performance is a weak LD for the additive-by-additive effects; while for the additive effect of a gene, the LD depends on the genetic distance between the gene and the linked marker, for the epistatic effect of a pair of genes, the LD depends on the product of the genetic distance between each gene of the pair and their linked markers, which may result in poorer predictive performance when relationship in reference population are lower.

In the literature, the value of including epistasis in GP has been population dependent and has varied among studies. While in some studies the PA increased (Heslot et al. 2012; He et al. 2016), in others, it changed very little (Jarquín et al. 2014) or even decreased (Lorenzana and Bernardo 2009). Increases in PA ranging from 4 to 25% have been found for random folds CV (fivefold or tenfold) when shifting from additive to additive plus epistatic effects models in wheat (Crossa et al. 2010; Heslot et al. 2012; Jiang and Reif 2015; He et al. 2016), which agrees with the range of improvement found in our study for the LOO CV. Recently, Schrauf et al. (2020) found a better PA for non-additive models even when non-additive variance was expected to be low. They attributed this improvement to a better capacity of epistatic models to capture additive variance (of causal loci) associated with non-additive apparent effects (on markers) at low marker densities (“Phantom epistasis”). These authors have warned on the risk of over-interpretation of the biologically functional meaning of estimated statistical parameters. While a straightforward biological interpretation is to relate the highest PA of epistatic models to an underlying genetic architecture of substantial additive-by-additive epistasis, it could also reflect “Phantom epistasis” due to incomplete LD due to low marker density. Contrasting these results, Lorenzana and Bernardo (2009) using a fivefold CV found a poorer performance for predictions when the model accounted for additivity and epistasis in comparison with a model accounting only for additivity. The discrepancies among the results found may be explained by differences in marker density, the level of additive-by-additive epistasis among the evaluated populations. Forneris et al. (2017) explored the effect of including epistasis in the evaluation model (knowing the causal mutations), and they reported that including epistasis in the models when there was none led to lower prediction accuracies.

Beyond the discussion of whether the improvement in PA comes from a real reflection of additive-by-additive epistasis or from apparent epistasis, this does not undervalue the potential of epistatic models to improve GS. Therefore, the statistical advantage of improving GS is recognized and the use is encouraged. In addition, the expert knowledge about the genetic architecture of the trait as well as the type of population and species may be relevant factors to determine the potential of including epistasis in GS.

### Inflation of variance and bias

The test for variance inflation in the predicted genetic effects, calculated as the regression of estimated values with whole information on estimated with partial information ($b_{w,p}$), led to regression coefficients close to 1 for the LOO CV, which means that none of the proposed models had a significant under- or over-dispersion in their predictions. Note that the LOO CV represents an optimal scenario due to the use of the largest possible reference population for predictions, and therefore, under- or over-dispersion in predictions of genetic values is in general not observed. In the LSO, values of $b_{w,p}$ lower than 1 were observed for predictions of both genetic effects (GEBVs and GEEVs), indicating over-dispersion of genomic predicted values. Particularly, predictions of epistatic values (GEEVs) for the I + G_A_ + G_AA_-model had the lowest $b_{w,p}$ value ($b_{w,p}$ = 0.70), suggesting that the epistatic predictions were more sensitive to the lack of information in the reference population. The bias ($\mu_{wp}$) of predictions had coefficients close to 0 for GEBVs and GEEVs in both CVs utilized; it indicates that unbiased genomic values were reached for all proposed models.

### Correlation between G-BLUP and NOIA estimates

We found that Pearson’s correlation between GEBVs from I + G_A_-model and I + G_A_ + G_AA_-model was high (0.94) compared to the correlation between GEBVs and genomic estimated epistatic values (0.65). Accordingly, differences were also evidenced in a change of ranking between lines with superior additive value (based on GEBVs) and lines with superior total genetic value (based on GEBVs plus genomic estimated epistatic values), indicating that the use of I + G_A_ + G_AA_-model to predict lines with higher total genetic value led to a different selection of candidate lines than using the I + G_A_-model. These differences could be exploited by addressing the selection of crossing parents based on the I + G_A_-model predictions and commercial varieties based on the I + G_A_ + G_AA_-model predictions.

As reflected by the LOO CV, the current study confirms the potential of increasing the PA for total genetic merit by including epistasis in GS models. Importantly, the differences found for I + G_A_-model and I + G_A_ + G_AA_-model must not be interpreted as exclusive for the NOIA parametrization since other codings such as for EG-BLUP based on Su et al. (2012) or Martini et al. (2016) are equivalent in the current scenario (Joshi et al. 2020). Further studies are required to: (i) investigate the influence of genetic relationships on the performance of epistatic predictions and develop CVs schemes that allow to capitalize the benefit of epistatic models in wheat breeding programs, (ii) develop breeding programs that consider more elaborate mating schemes in order to improve the genetic relationships between breeding cycles, and (iii) develop a GP model in which the inclusion of pairwise interaction effects has minimal impact on the estimates of additive effects and their variance.

## Conclusions

In this research, we found that the orthogonal partition of genetic variances into additive and additive-by-additive epistatic effects was not possible. Nevertheless, including additive-by-additive epistasis in a genomic prediction model increased predictive ability for total genetic merit significantly (16.5%) compared to an additive genomic-based model in a leave-one-line-out cross-validation. The advantage of including epistasis in predictive ability was not observed for a leave-one-breeding-cycle-out cross-validation. Further studies are required to: (i) investigate the influence of genetic relationships on the performance of epistatic predictions and develop CVs schemes that allow to capitalize the benefit of epistatic models in wheat breeding programs, (ii) develop breeding programs that consider more elaborate mating schemes in order to improve the genetic relationships between breeding cycles, and (iii) develop a GP model in which the inclusion of pairwise interaction effects has minimal impact on the estimates of additive effects and their variance.

## Supplementary Information

Below is the link to the electronic supplementary material.

Supplementary file1 (DOCX 78 KB)

## Data Availability

The datasets analyzed during the current study are available in the Harvard dataverse public repository at the following link: https://dataverse.harvard.edu/dataset.xhtml?persistentId=doi:10.7910/DVN/ULGTGT.

## Abbreviations

ADD: Predicted additive values
CV: Cross-validation
DK: Denmark
EG-BLUP: Extended genomic best linear unbiased prediction
EPI: Predicted epistatic values
G-BLUP: Genomic best linear unbiased prediction
GEBV: Genomic estimated breeding value
GEEV: Genomic estimated epistatic value
GP: Genomic prediction
GS: Genomic selection
HWE: Hardy–Weinberg equilibrium
LD: Linkage disequilibrium
LE: Linkage equilibrium
LOO: Leave-one-line-out
LSO: Leave-one-breeding-cycle-out
MAF: Minor allele frequency
NOIA: Natural and orthogonal interactions approach
PA: Predictive ability
RD: Relative difference
SNP: Single nucleotide polymorphism
VC: Variance components
WGR: Whole genome regression
