# Supplementary material for: Improvement of genomic prediction in advanced wheat breeding lines by including additive-by-additive epistasis
Source: Theor Appl Genet. 2022 Jan 1;135(3):965–78. doi: 10.1007/s00122-021-04009-4 (PMC8942904; doi:10.1007/s00122-021-04009-4)
Supplement: Supplementary file 1 — Supplementary file1 (DOCX 78 KB) [file 122_2021_4009_MOESM1_ESM.docx]

**Supplementary material 1**

**
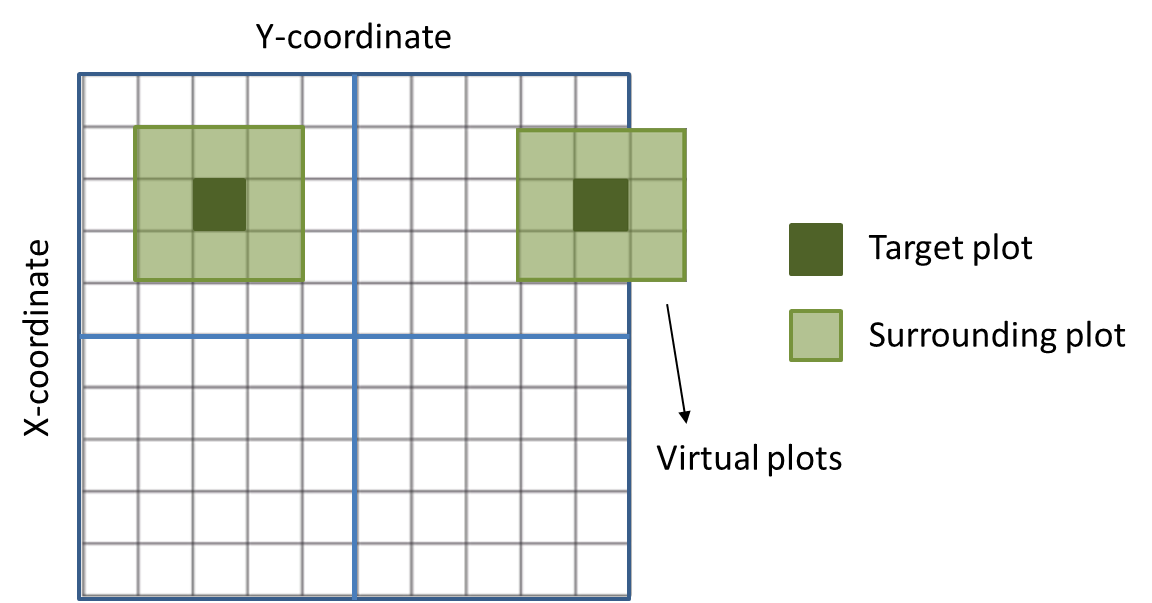
**

**Figure S1.** Schematic representation of spatial information in a field trial. The central squares (dark green) represent the target plot that together with the eight surrounding plots (light green squares) are included in the model to correct by the special variability of the field. For every observation, it has been assigned one central plus eight surrounding plots. For the plots outside the borders of the trials (blue line limit), virtual plots have been assigned in order to complete the eight surrounding plot. Therefore, the spatial effects on an individual plot is the sum of effects with the square centered on the plot itself plus the effects of eight surrounding plots with a square centered on those plots.
